# Acute effects of selective serotonin reuptake inhibitors on cerebral glucose metabolism and blood flow

**DOI:** 10.1038/s41398-026-03849-2

**Published:** 2026-02-03

**Authors:** Leo R. Silberbauer, Murray B. Reed, Gregor Gryglewski, Matej Murgaš, Lukas Nics, Godber Mathis Godbersen, Thomas Stimpfl, Andreas Hahn, Marcus Hacker, Rupert Lanzenberger

**Affiliations:** 1 Department of Psychiatry and Psychotherapy, Medical University of Vienna, Vienna, Austria; 2 Comprehensive Center for Clinical Neurosciences and Mental Health, Medical University of Vienna, Vienna, Austria; 3 Department of Biomedical Imaging and Image-guided Therapy, Division of Nuclear Medicine, Medical University of Vienna, Vienna, Austria; 4 Department of Laboratory Medicine, Medical University of Vienna, Vienna, Austria

**Keywords:** Psychiatric disorders, Neuroscience

## Abstract

Selective serotonin reuptake inhibitors (SSRIs) are widely prescribed antidepressants, though their mechanisms of action beyond serotonin transporter (SERT) blockade remain unclear [1]. As previous work on BOLD signal changes remain equivocal, pharmacological multimodal neuroimaging of energy demands and blood flow (CBF) holds promise due to increased specificity of these signals. This may advance the understanding of the involved pharmacodynamic mechanisms and guide treatment strategies of highly prevalent neuropsychiatric disorders. We combine new techniques of functional positron emission tomography (fPET) with high temporal resolution (3 seconds) using [^18^F]FDG and simultaneously acquired pseudo-continuous arterial spin labelling (pcASL). Thus, we aimed for a highly quantitative assessment of changes in brain activation following an intravenous SSRI challenge using a randomized, placebo-controlled, double-blind study design. We demonstrate acute drug induced changes in glucose metabolism (K_i_) in serotonergic projections, i.e. the striatum and the occipital cortex in 16 healthy volunteers (7 females). In an exploratory analysis, acute effects were observed in the dorsal raphe nucleus. We did not observe corresponding changes in CBF, which suggests that observed SSRI effects are specific to brain energy demands. Our results complement the existing literature on the acute pharmacological effects of SSRIs by providing insights in specific aspects of neuronal activation. Moreover, our findings expand upon the results of existing BOLD fMRI studies and, thus, support the application of this pharmacological neuroimaging protocol in psychopharmacological research.

## Introduction

Selective serotonin reuptake inhibitors (SSRIs) are considered as first-line pharmacological treatment for highly prevalent psychiatric disorders such as major depressive disorder (MDD) [2]. However, up to 60% of patients do not respond sufficiently to initial treatment [3] and mechanisms of action beyond the initial blocking of the serotonin transporter (SERT) remain poorly understood.

Pharmacological neuroimaging represents an important tool to assess acute drug effects in vivo and might therefore aid in the identification of predictors of treatment response. So far, the majority of studies investigating acute SSRI effects on brain activation have used functional magnetic resonance imaging (fMRI) utilizing the blood oxygenation level dependent (BOLD) signal [4–6]. Increases in the BOLD signal after intravenous challenge with the SSRI citalopram were reported in regions implicated in depressive pathophysiology including the anterior cingulate cortex and the caudate nucleus [4]. Similar results were obtained in a subsequent trial [6], however, results were not replicated in a larger population when multiple modeling approaches and appropriate correction of multiple testing were applied [5]. Equivocal results may be explained by the non-stationary nature of the BOLD signal. The relativity of the measure hampers the establishment of a baseline to which data acquired after drug challenge can be compared [7]. Moreover, the BOLD signal is a composite of cerebral blood flow (CBF), volume and oxygenation, and thus, only allows for the indirect assessment of neuronal activation [8].

Therefore, assessing neurophysiological underpinnings of acute drug effects more directly holds promise to advance the understanding of the molecular mechanism of action of SSRIs. Functional positron emission tomography (fPET) with [^18^F]FDG as bolus plus constant infusion [9] represents a novel technique that facilitates the dynamic assessment of drug-induced brain energy demands. Its outcome parameter, the net influx constant (K_i_), is considered as a more direct marker of neuronal activation, when compared to the BOLD signal, due to its specificity for glucose metabolism. Therefore, it reflects a single and clear defined physiological process, and thus, eases the interpretation of results. Recent advances in filtering techniques [10] allow for the reconstruction of high-temporal resolution fPET data with frames of 3 seconds [11] enabling a fine-grained analysis of pharmacological effects on metabolism. Moreover, fPET fully exploits the potential of hybrid PET/MR scanners as it allows for the simultaneous acquisition of MRI data [12]. As a temporally stable signal with high test-retest reliability [13, 14] that allows for a straightforward physiological interpretation [15, 16], CBF measured via arterial spin labeling (ASL) provides complementary insights to fPET with [^18^F]FDG. However, studies on acute SSRI effects on CBF remain limited and lack a placebo control [17–19].

In this work, we aimed for a highly quantitative assessment of SSRI-induced changes in neuronal activation by combining [^18^F]FDG fPET and ASL in a randomized, placebo-controlled, double-blind study design. This overcomes the limitations and contradictory results of pharmacological BOLD imaging as outlined above. As spatiotemporal effects of acute SSRI challenge are currently unknown, intersubject correlations were used to allow for the assessment of drug effects independent of a predefined model. Specifically, the simultaneous quantification of glucose metabolism and CBF enables the complementary characterization of neuronal activation by disentangling distinct metabolic underpinnings of acute SSRI effects.

## Methods

### Participants and study design

Sixteen healthy volunteers (mean age ± SD = 26.9 ± 8.2, 7 female) were enrolled in this study, each undergoing two hybrid [^18^F]FDG fPET/MRI scans. A pharmacological challenge with the SSRI citalopram or placebo was administered during scans in a randomized, cross-over, double-blind study design.

General health was assessed via a medical examination comprising medical history, physical examination, electrocardiogram and routine laboratory parameters. The Structured Clinical Interview for DSM-IV for Axis I disorders (SCID-I) was used to exclude the presence of any previous or current psychiatric disorder. Exclusion criteria comprised chronic medical conditions, psychiatric disorders, current and previous substance use disorder, current and previous psychopharmacological treatment and contraindications for PET/MR scans such as implants, claustrophobia and previous radiation exposure. Urine drug tests were performed at screening. With female participants, urine pregnancy tests were performed at screening and before each scan. All participants provided written informed consent and received financial reimbursement for their participation. This study was approved by the ethics committee of the Medical University of Vienna (EK 1307/2014) and was carried out according to the Declaration of Helsinki. This investigation is part of a larger study that was registered before the start of recruitment at clinicaltrials.gov (NCT02711215). Subjects were recruited between September 2019 and September 2020.

### PET/MR scanning procedures

Subjects were required to fast (except for water intake) for a minimum of 5.5 h before administration of the radioligand [20]. A cannula was inserted in the radial artery for arterial blood sampling. Another cannula was inserted in a cubital vein of the opposite arm for the administration of the radiotracer [^18^F]FDG and study medication (citalopram or placebo). Synthesis of [^18^F]FDG and was carried out according to an established protocol [21]. The radiotracer was applied as bolus (1020 kBq/kg/min, 1 min) plus constant infusion (83.3 kBq/kg/min, 49 min) using a perfusion pump (Syramed µSP6000, Arcomed, Regensdorf, Switzerland), which was placed in an MR shield (UniQUE, Arcomed). All scans were performed on a hybrid 3 T PET/MRI scanner (Siemens mMR Biograph, Siemens Healthineers, Germany) installed at the Department of Biomedical Imaging and Image-guided Therapy, Division of Nuclear Medicine, Medical University of Vienna. A structural image was acquired with a T1-weighted MPRAGE sequence prior to radiotracer administration with the following parameters: (TE/TR = 4.21/2200 ms, TI = 900 ms, flip angle = 9 °, matrix size = 240×256, 160 slices, voxel size = 1 ×1 x 1 mm + 0.1 mm gap, 7.72 min). Subsequently, fPET acquisition (50 min) started 1 minute prior to radiotracer application as previously described [12]. The study medication was diluted in saline and provided in syringes of 50 ml. Twenty minutes after fPET baseline data acquisition, the double-blind pharmacological challenge with either citalopram 8 mg or saline was performed as a continuous infusion over 8 min via an automated syringe pump.

A pseudo-continuous arterial spin labeling (pCASL) sequence with background-suppressed 3D GRASE readout was started 5 min before drug challenge for a total of 35 min. Sequence parameters were selected based on the protocol described by Wang et al. [22]. The labeling plane was positioned 90 mm below the imaging slices, and background suppression was enabled. The post-labeling delay (PLD) was set to 1800 ms, with a labeling duration of 1500 ms. Imaging was performed with an isotropic resolution of 2.5 × 2.5 × 2.5 mm^3^, a repetition time (TR) of 4100 ms, and an echo time (TE) of 36.76 ms. A separate M_0_ image was acquired as the first volume for quantification.

### Blood sampling

Individual fasting glucose levels were assessed before scans (Glu_plasma_, triplicate measurement). Arterial samples were drawn at 3, 4, 5, 18, 22, 25, 28, 32, 40, 50 min after start of the tracer application. Samples drawn at 3, 4, 5, 18, 25, 32, 40 and 50 min were used for quantification of glucose metabolism following a previously established protocol [12]. Arterial blood samples were processed as described previously [9]. Whole-blood and plasma activity was measured in a gamma counter (Wizard2, Perkin Elmer). Whole-blood data were linearly interpolated to match PET frames and multiplied by the average plasma-to-whole-blood ratio to obtain the arterial input function.

Successful pharmacological drug challenge could be verified by the assessment of citalopram concentration from plasma levels drawn at 22, 25, 28, 32, 40, 50 min after start of radiotracer application in all participants. Prior to analysis of blood plasma concentration, the samples were stored at -80 °C. Citalopram concentrations were assessed with MassTox® TDM Serie A test-kits (Chromsystems, Gräfeling, Germany) and liquid chromatography-tandem mass spectronomy (LC-MS/MS) at the Department of Laboratory Medicine, Medical University of Vienna.

### Quantification of glucose metabolism

Preprocessing was performed using established protocols as detailed in previous reports [12, 23] unless specified otherwise and included attenuation correction using a pseudo-CT approach based on structural MRI data [24]. fPET images were reconstructed to frames of 3 seconds (matrix size = 344 ×344, 127 slices) to allow for the investigation of fine-grained pharmacological effects and comparison with previous BOLD studies. This was followed by head movement correction (quality = 1, registration to mean image) and spatial normalization to MNI space. A dynamic non-local means filter (NLM) [10] was used to increase the low signal to noise ratio inherent to high-temporal resolution fPET data as less radioactive counts are registered in short frames [11]. The gray matter mask was delineated using the gray matter tissue probability map in SPM 12, applying a threshold ( > 0.1) to include only voxels with a high probability of belonging to gray matter. Considering the previously contradicting findings of an acute pharmacological SSRI challenge and the unknown spatio-temporal activation pattern [4, 5], modelling of drug effects was performed with a novel approach combining two different methods:

a) In the first approach, an average baseline [^18^F]FDG time activity curve (TAC) across all gray matter voxels was modeled by a third order polynomial as in our previous work [9, 12]. The TAC was fitted for each single voxel from minute 5 to 20 with a general linear model. The initial 5 min were discarded, as done in previous work, to improve model fit by excluding potential bias from pronounced initial radiotracer uptake [25]. For each voxel, the fitted TAC was extended to the full time course with the obtained parameters and subtracted from the original TAC. This yields a time course that ideally only reflects challenge-induced changes in glucose metabolism starting at minute 20, i.e., after initiation of the pharmacological challenge. Pairwise intersubject correlations (ISC) were performed on the verum scan of each participant [26, 27]. Specifically, for each voxel the obtained time course was correlated between pairs of subjects. To compute the correlation map for one subject, the average across all other subjects was calculated [26, 27]. Next, the grand mean across all subjects and voxels was computed and subtracted from each individual correlation map. A subsequent one-sample t-test in SPM12 yielded significant clusters, where the intersubject correlation is significantly different from the average correlation (p < 0.05 FWE-corrected cluster level, after p < 0.001 uncorrected voxel level). Within significant clusters, the time courses were plotted, which identified a linear increase of glucose metabolism during pharmacological challenge. The drug-induced changes were therefore modelled as a linear function for the verum and the placebo scans, with subsequent comparison for each of the identified clusters.

b) A second confirmatory approach was used to directly compare effects of verum vs. placebo at the whole-brain level. Proceeding from the first approach, intersubject correlations were also carried out for the individual placebo scans. The maps of verum and placebo were then directly compared in SPM12 using a paired t-test. These two approaches carry slightly different but complementary information. The focus of the first one (a) is to identify brain regions with verum-specific increases in glucose metabolism, but comparison to placebo is done post-hoc. On the other hand, the second approach (b) identifies brain regions based on the difference between verum and placebo, but it does not rule out that effects are driven by placebo. Thus, brain regions showing significant results in both approaches represent a) verum-specific effects, which b) are also significantly different from placebo at the whole-brain level.

The Gjedde-Patlak plot was used for quantification of glucose metabolism resulting in the net influx constant K_i_ (min^-1^). This parameter reflects the combination of the individual rate constants: K_i_ = K1*k3/ (k2 + k3). Like CMRGlu, K_i_ serves as an index of glucose metabolism but is more reliable since it is unaffected by variations in individual plasma glucose levels [14].

In an exploratory analysis, we assessed acute citalopram effects on glucose metabolism in the dorsal raphe nucleus (DRN). This midbrain region, as the origin of serotonergic projections, plays a central role in the acute pharmacological effects of SSRIs [28, 29]. Previous PET studies have further highlighted its significance, particularly in predicting treatment response [30, 31]. Thus, acute citalopram effects on glucose metabolism in the dorsal raphe nucleus (DRN) were assessed. Here, the average baseline [^18^ F]FDG TAC was modelled across gray matter voxels within the brainstem that was delineated with the Harvard-Oxford atlas as provided in FSL. The small size of the raphe region makes its precise delineation a well-known methodological challenge in neuroimaging studies [32]. To address these methodological limitations, we applied an individualized adjustment procedure. High visual agreement between mean baseline [^18^F]FDG uptake and DRN coordinates as provided from *Cauzzo* et al. was observed [33]. To account for interindividual variability, the DRN was delineated individually by shifting the center coordinate of the DRN template from *Cauzzo* et al. [33] to the nearest local maximum of tracer uptake separately for each individual. Drug effects were modelled as a linear function and quantification of glucose metabolism was performed as described above. Based on the assumption that SSRI-induced increases in synaptic serotonin concentration accumulate over a period that exceeds the duration of the infusion [5] and preclinical evidence of slower increases in extracellular serotonin following drug challenge in the raphe nuclei [34], drug effects were assessed step-wise beyond the infusion period, i.e. minute 20 to 28, in intervals of 5 min.

### Quantification of cerebral blood flow (CBF)

Processing of pcASL data was performed using the ASLtbx in SPM12 [35]. The equilibrium magnetization of the brain (M_0_) was extracted from the pcASL time series [22]. The M_0_ image and ASL data were coregistered to the structural T1 image. Non-brain voxels were removed using the brain extraction tool implemented in FSL [36] and the resulting mask was applied to the images of the time series. The data were temporally filtered using the ASLtbx function. Finally, data were smoothed with an 8 mm Gaussian kernel. Quantitative perfusion values from the label/control ASL images were calculated as follows:

$${CBF}=\frac{{\lambda \Delta {MR}}_{1a}}{{2\alpha M}_{0}\{\exp \left(-\omega {R}_{1a}\right)-\exp [-\left(\tau +w\right){R}_{1a}]\}}$$

With λ being the blood-tissue water partition coefficient ( = 0.9 ml/g), ΔM the difference between label and control images, R_1a_ the longitudinal relaxation rate of blood ( = 0.6 s^-1^), α the tagging efficiency ( = 0.8), ϖ the post-labeling delay of 1800s and τ the labeling pulse duration ( = 1.5 sec). Finally, time series were saved, normalized and masked again using the whole brain mask created before. Intersubject correlations were calculated as described for fPET data. For significant clusters, the average CBF after the challenge was calculated for comparison between verum and placebo.

### Statistical analysis

To assess differences between citalopram and placebo, repeated measures ANOVA (rmANOVA) was calculated for each of the two outcome parameters (K_i_ and CBF) in separate models for significant clusters obtained from the intersubject correlations. This was followed by post-hoc two-tailed t-tests for each brain region. Correction for multiple comparisons was performed using the Bonferroni-Holm procedure at alpha = 0.05.

## Results

Successful drug challenge was verified by citalopram plasma levels in all subjects. Peak citalopram plasma levels ranged from 47.1 to 169.8 ng/ml (mean ± sd = 90.5 ± 29.63).

### Glucose metabolism

**Fig. 1:**
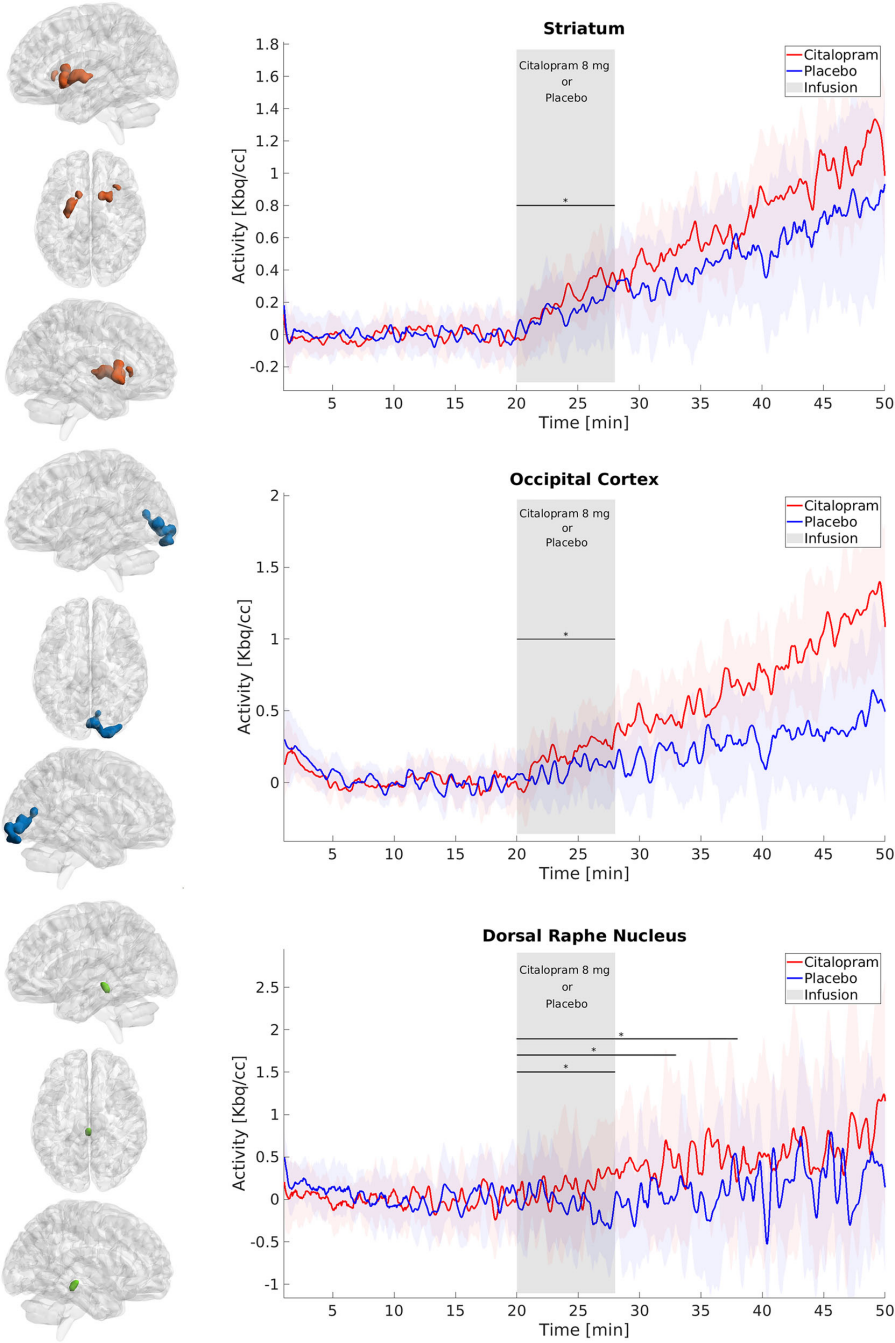
Glucose consumption dynamics within significant clusters and the dorsal raphe nucleus. Significant clusters from intersubject correlations of the citalopram scan (approach a) for cerebral glucose metabolism (Ki, p < 0.05 FWE-corrected cluster level, following p < 0.001 uncorrected voxel level) (left column). Glucose consumption is displayed for citalopram and placebo scans in significant clusters (right column). Shaded bands denote the standard error of the mean for each line. X- axis indicates minutes after initiation of [^18^ F]FDG application. The pharmacological challenge was administered intravenously over 8 min (grey field).

For approach a, higher than average inter-regional correlations were observed in the occipital cortex, the hippocampus, the dorsolateral prefrontal cortex, the cerebellum, the striatum, the temporal superior and precentral gyrus (p_FWE_ < 0.05 cluster level). rmANOVA showed a significant main effect for substance (p < 0.05) and ROI (p < 0.001) and a significant interaction effect of substance*region (p < 0.001). Post-hoc t-tests revealed a statistically significant difference in K_i_ between citalopram and placebo in the occipital cortex and the striatum (both p_Bonf-Holm_ < 0.05) with citalopram showing stronger increases in K_i_ as compared to placebo (Fig. 1). Approach b showed significant differences in the occipital cortex, striatum, hippocampus, precentral gyrus as well as inferior parietal and middle temporal cortices. Of those, only the first four regions were attributed to verum effects as per approach a (see Figure S1 for direct comparison). Also, the hippocampus did not withstand correction for multiple comparisons in a. Regarding the precentral gyrus, approaches a and b covered non-overlapping subparts, namely the face region (z = 20-40 mm MNI space) and the hand region (z = 50-58 mm), respectively. Thus, interpretations will be limited to the occipital cortex and striatum.

In the whole-brain analysis, no significant effects were detected in the raphe region. However, exploratory analyses revealed a statistically significant difference in glucose metabolism between citalopram and placebo in the DRN (p < 0.05). Modeling the drug effect as a linear function over the duration of the infusion revealed a significant reduction in glucose metabolism during placebo administration (p < 0.05), whereas trend-wise increases were observed for citalopram administration (p = 0.052). When extending the analysis intervals in steps of 5 min, significant differences between conditions emerged for the intervals 20–33 min and 20–38 min, each driven by a significant increase in glucose metabolism under citalopram, but not placebo. With further extension of the intervals (20–43 min, 20–48 min, and 20–50 min, i.e., end of scan), no significant between-condition differences were observed; however, this was attributed to significant increases in glucose metabolism under both citalopram and placebo.

### CBF

Significant clusters from intersubject correlations revealed higher than average inter-regional associations during citalopram in the left superior temporal pole, left inferior temporal pole, right anterior insula, right posterior insula, right superior occipital cortex, calcarine and the cuneus (p_Bonf-Holm_ < 0.05 cluster level). rmANOVA, however, revealed no significant region-by-substance interactions.

## Discussion

This hybrid pharmacological [^18^F]FDG fPET/MRI study assessed the acute effects of an intravenous citalopram infusion on cerebral glucose metabolism and blood flow. Significant effects of citalopram on cerebral glucose metabolism were observed in serotonergic projection areas, i.e., the striatum and the occipital cortex. Exploratory analyses revealed significant effects of citalopram on glucose metabolism in the raphe region. The lack of corresponding changes in CBF indicates that SSRI effects on metabolism cannot be explained by simple changes of blood flow but are specific to energy demands.

The effects of acute SSRI challenge emerge from the intricate interplay of two opposing processes, both instigated by the increase of extracellular serotonin concentrations: Reduced serotonergic cell firing via inhibitory presynaptic 5-HT_1A_ autoreceptors in the midbrain raphe region [37, 38] and net effects of local increases in extracellular serotonin concentrations in serotonergic projection areas (dependent on 5HT receptor and transporter density, see Figure S2) [39, 40]. High SERT expression in the raphe is well established using PET and SERT specific radioligands [41, 42]. Our [^18^F]FDG fPET study demonstrates acute SSRI effects on brain energy demands in the DRN for the first time in healthy human subjects in vivo. Previous electrophysiological studies demonstrated suppression of neuronal firing activity in the DRN within minutes after systemic administration [43]. The observed drug effect aligns with these findings and previous data on the rapid distribution kinetics of citalopram [44]. Immediate effects are further supported by [^11^C]DASB PET data indicating rapid changes in SERT binding after drug challenge [5, 45]. Drug effects in the DRN beyond the infusion period may be explained by slower distribution kinetics of citalopram in the raphe nuclei [34] and sustained accumulation of drug concentration [5]. The absence of significant differences between citalopram and placebo from minute 38 onwards in the DRN may be attributable to the small size of the region, which increases signal variability and thereby reduces statistical power to detect effects. However, the temporally restricted effect in the DRN is at odds with preclinical data showing more prolonged suppression of neuronal firing, which may be attributed to complex human feedback mechanisms [46].

Our finding of increased glucose metabolism in serotonergic projection areas should be interpreted in the context of the uncertainty regarding the acute effects of SSRI administration on local serotonin concentrations. Preclinical studies indicate a rapid reduction of serotonin levels in projection areas [43] due to autoreceptor-mediated feedback inhibition in the raphe, which may reduce terminal release in cortical projection regions. In humans, earlier PET studies have similarly reported cortical decreases in serotonin concentrations, though only around 3 h after oral SSRI administration [47]. Notably, this includes the occipital cortex, where we found an acute increase in glucose metabolism during the infusion. While we observed changes both in the raphe and in projection areas, the available evidence suggests that the effects in projection regions are unlikely to be explained by altered serotonergic activity in the raphe and concomitant serotonin release [48]. Rather, they are more plausibly explained by local reuptake inhibition and its downstream impact on region-specific cellular and receptor profiles, with the [¹⁸F]FDG signal generally considered to predominantly reflect postsynaptic energy demands related to the restoration of ionic gradients following synaptic activity [23, 49].

We detected increased glucose metabolism in the striatum, a region linked to antidepressant SSRI effects due to serotonin´s role in reward processing [50, 51]. The striatum receives strong serotonergic innervation from the DRN [52] and displays high SERT and 5-HT_1B_ receptor expression (see Figure S2) [53]. Our findings suggest that increased glucose metabolism in the striatum during drug challenge is associated with local increases in extracellular serotonin concentration due to striatal SERT blockade rather than changes in raphe activity [48]. This hypothesis aligns with previous electrophysiological studies demonstrating increases in local serotonin concentration in the striatum following citalopram challenge [54]. However, the mechanisms linking increased extracellular serotonin concentration to increased brain energy consumption remain to be elucidated. GABAergic medium spiny neurons, the largest neuronal population in the striatum, may be responsible for drug-induced activation, potentially through serotonergic autoreceptors or receptors located on interneurons [55]. Additionally, efferents from the anterior cingulate cortex (ACC) and prefrontal cortex (PFC) may play a role [40]. Increases in striatal glucose metabolism align with previous human pharmaco-fMRI studies showing SSRI-induced increases in the BOLD response in this region [4, 6]. Moreover, our finding of acute increases in striatal glucose metabolism can be conceptualized within the framework of antidepressant effects on resting-state networks. Pioneering work using trimodal PET–MRI–EEG has demonstrated that glucose metabolism is tightly coupled with functional and electrophysiological signatures, as well as with the underlying excitatory/inhibitory (E/I) balance of core networks such as the default mode and salience networks [56–58]. Thus, SSRI-induced changes in striatal glucose metabolism may be interpreted as a modulation of the frontostriatal salience network, which was recently shown to be expanded in depression [59].

The observed increase in glucose metabolism in the occipital cortex following acute SSRI challenge may seem counterintuitive in the context of depressive pathophysiology and antidepressant mechanisms. The significant cluster overlaps with the primary visual cortex, a region with the most complex neural circuitry among all sensory systems [60]. This area displays a unique serotonin receptor distribution, with low 5-HT_1A_ and high 5-HT_2A_ receptor expression (Figure S2) [53]. Opposing functional effects of these major receptor subtypes are well established [61, 62]. While serotonin´s inhibitory effects, mediated by the 5-HT_1A_ receptor, likely dominate most cortical regions [63], the unique receptor distribution in the visual cortex may result in neuronal activation due to SERT-mediated increases in synaptic serotonin concentration and subsequent 5-HT_2A_ receptor activation. Increased glucose metabolism aligns with preclinical studies showing a serotonin-mediated shift in the E/I balance toward excitation in the visual cortex [64]. Serotonergic modulation of thalamocortical feedback loops may account for the absence of changes in glucose metabolism in other serotonergic projection areas [40]. Furthermore, the structural similarity between serotonin and the 5-HT_2A_ receptor agonist psilocybin suggests that citalopram-induced increase in neuronal activity within primary sensory areas may represent a cross-class unifying effect between classical antidepressants such as SSRIs and psychedelics that are commonly associated with altered sensory processing, e.g., visual hallucinations [65].

When comparing our results with previous pharmacological imaging studies using SSRIs, methodological differences should be considered. The reported effect of acute SSRI challenge on cerebral glucose metabolism is based on quantification of glucose consumption (K_i_) dynamics. This is facilitated by a constant supply of [^18^F]FDG during the entire scan duration, which allows the radioligand to bind according to drug-induced changes in energy. In contrast, previous [^18^F]FDG PET studies assessed glucose metabolism changes after SSRI challenge at a fixed time interval of at least 30 min post infusion, providing only a static snapshot of drug effects [66–68]. While previous BOLD fMRI studies investigating SSRI effects with the significant advantage of high temporal resolution inherent to this technique, the changes in the fMRI signal merely represent an indirect proxy for activation [69]. The herein employed high temporal resolution of 3 seconds for fPET [11] overcomes the limitations of the BOLD signal at a comparable temporal resolution. Direct comparison of the two techniques suggest a strong correlation between locally circumscribed stimulation-induced increases in glucose metabolism and blood oxygenation [12, 69–71]. Thus, convergence of glucose metabolism and the BOLD signal following neuronal activation may be assumed for the ease of interpretation of our results [4, 6].

In line with our results, *McKie* et al. reported increases in the BOLD signal in subcortical regions, including the striatum and the occipital cortex, alongside increases in frontal and temporal regions [4]. This work informed the design of subsequent pharmacological studies, including our investigation, which used an identical pharmacological challenge. No statistically significant effects on the BOLD signal were observed by *Gryglewski* et al. in a threefold larger sample size after appropriate correction of multiple testing [5]. Another study reported initial and most pronounced BOLD signal increases in the lingual gyrus of the occipital cortex, followed by dose-dependent spreading of activation in an occipito-frontal direction, including subcortical regions like the caudate [6]. Our study differs in that we combined two highly quantitative imaging modalities without assessing dose responsiveness. The applied 8 mg citalopram dose was chosen to ensure tolerability during scanning, though higher doses may produce more widespread metabolic effects.

We did not observe significant drug-induced CBF changes following citalopram challenge using a 3D pcASL sequence acquired simultaneously with fPET data. This metric displays higher test-retest reliability than the BOLD signal [13], a critical consideration given that scans in this study were performed on separate days. Our findings are at odds with preclinical data indicating close coupling between SSRI induced changes in blood flow and metabolism [72]. An interpretation that might reconcile these findings with our results is the directionality of the effect: While preclinical studies attributed decreases in blood flow to the SSRI-induced reduction in glucose metabolism, we observed an increased glucose metabolism following SSRI challenge that was not associated with changes in CBF. Moreover, our data support previous [^18^F]FDG fPET studies, which demonstrate that the fPET signal is not flow-sensitive [73]. Notably, interpretation is limited by the non-uniform temporal resolution of CBF and K_i_ data and the absence of evidence does not constitute definitive proof of absent CBF changes. In line with our results, no CBF effects were detected in a prior study using a nearly identical citalopram challenge and a time bin analysis [19]. Another study using oral citalopram challenge observed small CBF effects in SERT-rich regions, but results were not replicated between sessions in a within-subject cross-over design [74]. We argue that acute low-dose SSRI administration may not impact blood flow, supporting prior pharmacological imaging studies where a low citalopram dose did not alter the BOLD signal, which is largely driven by CBF [5]. Taken together, this study supports the assumption that metabolic changes are not driven by blood flow alterations, emphasizing metabolism as a direct measure of neuronal activation and offering insights into complementary aspects of neural activity. However, the inclusion of only healthy controls limits the interpretation of our results in the context of antidepressant efficacy. Our results provide a cornerstone for applying [^18^F]FDG fPET in psychopharmacological research. Given the close link between glucose consumption and the glutamatergic neurotransmitter system, our protocol may be used to assess NMDA-receptor antagonist ketamine effects in future studies. Furthermore, this imaging protocol may be extended to patient cohorts, as markers of individual treatment response are urgently needed. Importantly, the fPET approach may be used to assess drug-specific effects on glucose metabolism within a single measurement, as demonstrated in task-based studies [12].

In conclusion, we demonstrate that acute citalopram challenge alters glucose metabolism in the raphe region and serotonergic projection areas. Our study expands upon the results of existing BOLD fMRI research and suggests that SSRI-induced changes in neural activity are not solely driven by cerebral blood flow alterations.

## Supplementary information

Supplemental Material

## Data Availability

Raw data will not be publicly available due to reasons of data protection. Processed data can be obtained from the corresponding author with a data-sharing agreement, approved by the departments of legal affairs and data clearing of the Medical University of Vienna.
