# Supplementary material for: Acute effects of selective serotonin reuptake inhibitors on cerebral glucose metabolism and blood flow
Source: Transl Psychiatry. 2026 Feb 3;16:54. doi: 10.1038/s41398-026-03849-2 (PMC12873367; doi:10.1038/s41398-026-03849-2)
Supplement: Supplementary file 1 — Supplemental Material [file 41398_2026_3849_MOESM1_ESM.docx]

**Acute effects of selective serotonin reuptake inhibitors on cerebral glucose metabolism and blood flow.**

***Supplemental information***

***Contents***

**Supplemental Text.** Supplementary methods……………………………………………………………………..2

**Supplemental Figure S1.** Effects of citalopram on glucose metabolism……………………………………...….3

**Supplemental Figure S2.** Distribution of key players of the serotonergic system…..……………………………4

**Supplemental Figure S3.** Consort Flow Diagram….………………………………………...…..……………….5

**Supplemental References.** ……………………………………………………………..…………………………6

**Supplementary methods**

Sample size

Power analysis using G*Power determined that a minimum number of 12 subjects is required to detect significant differences in glucose metabolism between conditions (p< 0.05 two tailed, power = 80%), based on effect sizes from previous task-based fPET studies.

Randomization and blinding

Subjects were randomized using block randomization with variable-sized blocks of 4 to 14 subjects using Matlab 2018b (MATLAB version: 9.5.0.944444 (R2018b), Natick, Massachusetts: The MathWorks Inc.; 2018.). Within each block, subjects were evenly assigned to receive either placebo or citalopram at the first scan. Randomization was performed by an independent researcher of our group not involved in study conduction and analysis.

The double-blind study medication was prepared by the hospital pharmacy of the Vienna General Hospital and was provided in syringes containing either citalopram or placebo diluted in saline. The study physician was provided with a sealed envelope containing the disclosure of the agent in case of adverse events requiring unblinding. Premature unblinding was not necessary in any participant.


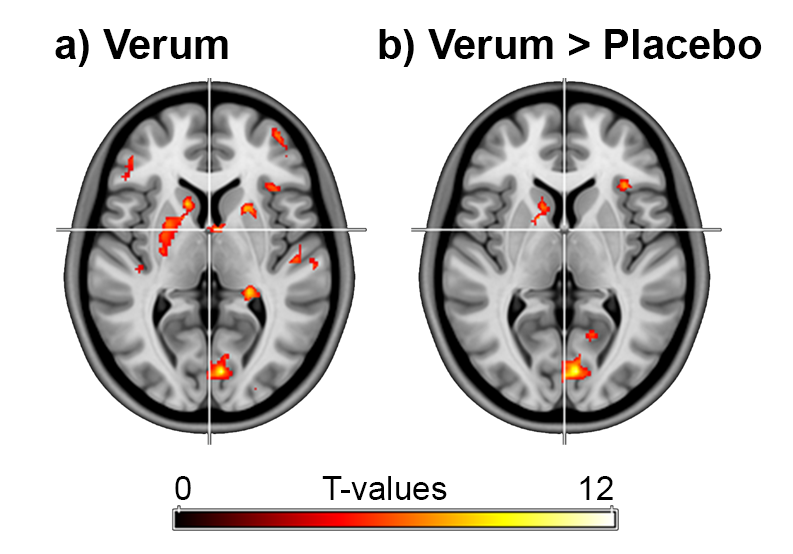


**Supplemental Figure S1. Effects of citalopram on glucose metabolism**. a) Approach a identified verum effects, with post-hoc comparison to placebo. b) Approach b shows the significant differences between verum and placebo at the whole-brain level, which does not per se distinguish if effects are driven by verum or placebo. Thus, the combination reveals effects a) specifically driven by verum which b) are also significantly different from placebo at the whole brain level. All p<0.05 FWE corrected cluster level, following p<0.001 uncorrected voxel level.


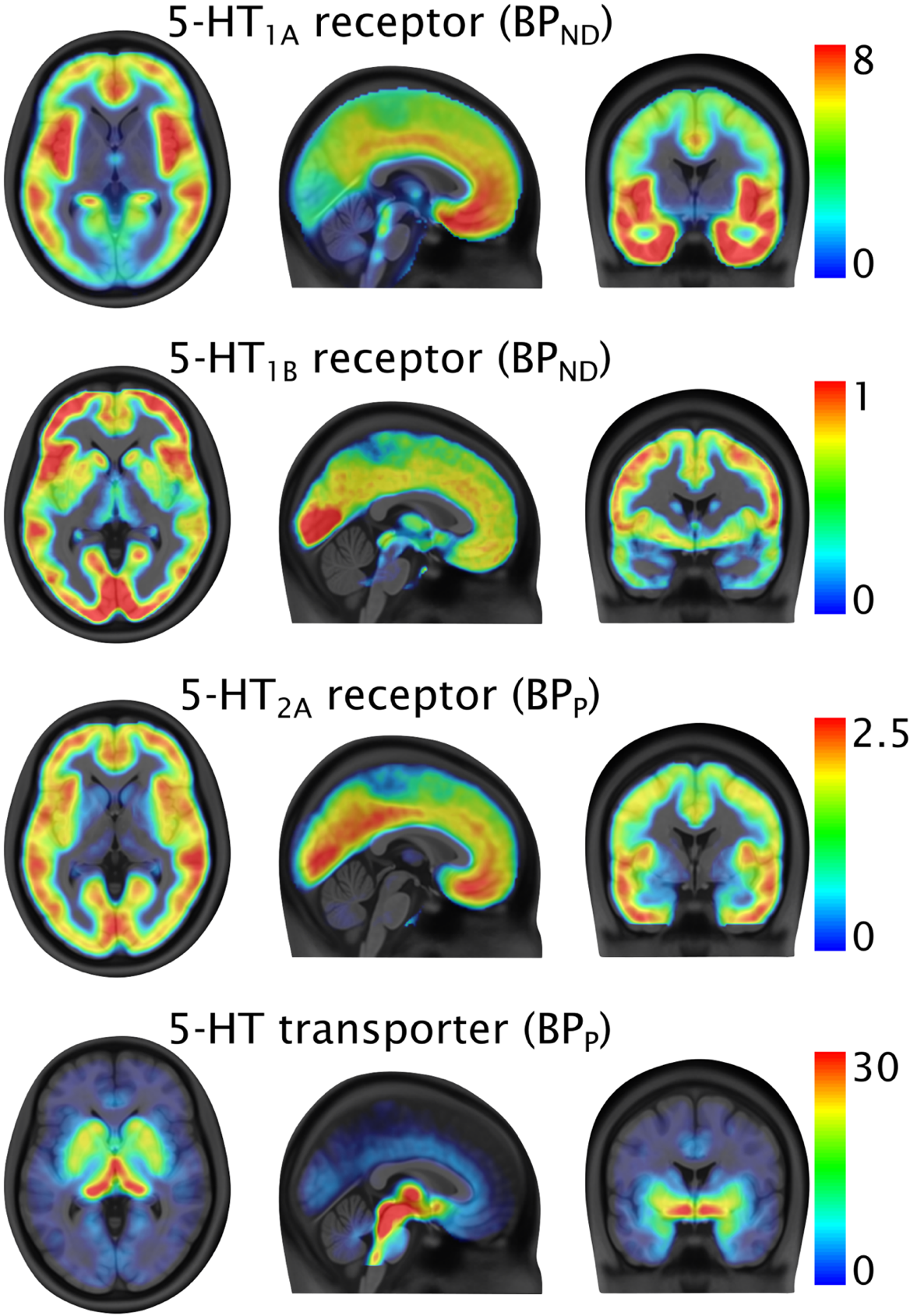


**Figure S2** **Distribution of key players of the serotonergic system.** Average binding potentials (BP) from healthy controls of the serotonin 1A receptor (5-HT_1A_), the serotonin 1B receptor (5-HT_1B_), the serotonin 2A receptor (5-HT_2A_) and the serotonin transporter (SERT) are displayed in transversal, sagittal and coronal planes overlaid on a MR template. Distinct receptor profiles in the striatum (high 5-HT_1B_ and SERT expression) and occipital cortex (low 5-HT_1A_, high 5-HT_1B_ expression) after infusion of 8 mg citalopram are associated with alterations in glucose metabolism. Binding potentials are derived from PET scans using [^11^C]WAY-100635 (5-HT_1A_), [^11^C]P943 (5-HT_1B_), [^18^F]altanserin (5-HT_2A_) and [^11^C]DASB (SERT) as published in Savli et al. [1].

**Supplemental Figure S3** Consort flow diagram

**REFERENCES**

1. Savli M, Bauer A, Mitterhauser M, Ding YS, Hahn A, Kroll T *et al.* Normative database of the serotonergic system in healthy subjects using multi-tracer PET. *NeuroImage* 2012; **63**(1)**:** 447-459.
